# Identification of the Transcription Co-Factor–Related Gene Signature and Risk Score Model for Osteosarcoma

**DOI:** 10.3389/fgene.2022.862803

**Published:** 2022-06-06

**Authors:** Zhijian Jin, Jintao Wu, Jianwei Lin, Jun Wang, Yuhui Shen

**Affiliations:** ^1^ Department of Orthopaedics, Ruijin Hospital, Shanghai Jiao Tong University School of Medicine, Shanghai, China; ^2^ Department of General Surgery, Ruijin Hospital, Shanghai Jiao Tong University School of Medicine, Shanghai, China

**Keywords:** transcription co-factors, osteosarcoma, risk score, gene signature, nomogram

## Abstract

Osteosarcoma is a malignant tumor with a poor prognosis. Nowadays, there is a lack of good methods to assess the prognosis of osteosarcoma patients. Transcription co-factors (TcoFs) play crucial roles in transcriptional regulation through the interaction with transcription factors (TFs). Many studies have revealed that TcoFs are related to many diseases, especially cancer. However, few studies have been reported about prognostic prediction models of osteosarcoma by using TcoF-related genes. In order to construct a prognostic risk model with TcoF-related genes, the mRNA expression data and matched clinical information of osteosarcoma were downloaded from the Therapeutically Applicable Research to Generate Effective Treatments (TARGET) database and the Gene Expression Omnibus (GEO) database. TARGET was used as a training set and GSE21257 from GEO was used as a validation set. Univariate Cox regression was performed to select 13 TcoF-related candidate genes, of which five genes (*LMO2*, *MAML3*, *MTF2*, *RBPMS*, and *SIRT1*) were finally used to construct the prognostic risk model by LASSO Cox regression analysis. The Kaplan–Meier (K-M) survival curves showed an obvious difference between high- and low-risk groups. The receiver operating characteristic (ROC) curves based on TARGET demonstrated that this risk model was credible (1-year AUC: 0.607; 3-years AUC: 0.713; 5-years AUC: 0.736). Meanwhile, the risk model was associated with immune cells and immune-related functions. By combining the risk score and clinical factors, the nomogram of osteosarcoma was assessed with a C-index of 0.738 to further support the reliability of this 5-gene prognostic risk model. Finally, the expression of TcoF-related genes was validated in different cell lines by quantitative real-time PCR (qRT-PCR) and also in different tissue samples by immunohistochemistry (IHC). In conclusion, the model can predict the prognosis of osteosarcoma patients and may provide novel targets for the treatment of osteosarcoma patients.

## Introduction

Osteosarcoma is a malignant tumor of mesenchymal origin, which is the most common primary sarcoma of bone in children and young adults (Picci, 2007; Anderson, 2016). Traditional prognostic determinants in osteosarcoma have included age, sex, tumor size, site, stage, and the response to chemotherapy. Although metastases and responses to chemotherapy are independent predictors of the prognostic condition of osteosarcoma, they usually appear at the late stage in the course of disease (Tomer et al., 1999; Bielack et al., 2002; Clark et al., 2008). Therefore, it has been a hot topic for researchers to target the diagnosis and prognosis of osteosarcoma. Unfortunately, little progress has been achieved since the underlying mechanisms are still unclear.

Transcription co-factor (TcoF) is defined as a protein that binds to a transcription factor (TF). It can modulate transcription initiation through a complex without direct interaction with DNA (Schaefer et al., 2011). As one of the major regulators of transcriptional regulation programs, it plays a crucial role in DNA loop maintenance and gene regulation. The protein activities of TcoFs can affect the expression of downstream target genes and help in maintaining the cell identity, while the dysfunction of TcoFs may lead to abnormal gene expression and induce the development of various diseases, suggesting that TcoFs may be potential therapeutic targets (Bai et al., 2011; Kim et al., 2020; Meyer et al., 2019; Russo et al., 2019; Tao et al., 2017; Ummarino, 2017; Zhang et al., 2022).

Recently, TcoFs have been found to be associated with a variety of tumors. Ramsdale et al. found that increased abundance of the TcoF c-JUN, which is activated by the JNK pathway, mediated both inherent and adaptive resistance to BRAF inhibitors and contributed to the metastatic potential of melanoma (Ramsdale et al., 2015). Megan et al. found that the embryonic TcoF LBH was a direct target of the Wnt signaling pathway in epithelial development and in aggressive basal subtype breast cancers (Rieger et al., 2010). The oncogenic function of LMO1 was first identified in T-ALL and neuroblastoma (Boehm et al., 1991; Wang et al., 2011). Also, the high expression of LMO1 in gastric cancer may be an indicator of poor prognosis (Sun et al., 2017). In addition, the overexpression of LMO4 was identified as a marker of poor prognosis in breast cancer (Sum et al., 2005). However, the roles of TcoFs in the diagnosis and prognosis of osteosarcoma have not been studied. Moreover, recent studies on TcoFs mainly focused on the role of a single gene in the development and prognosis of tumors.

To evaluate the potential of combined TcoF-related genes as biomarkers to predict the prognosis of osteosarcoma, we established a risk model by using five TcoFs and validated the performance of the model by survival analysis and multivariable Cox regression. Additionally, we assayed the expression of screened genes in osteosarcoma cell lines and tissues.

## Methods

### Data Collection

The mRNA expression data and matched clinical information of osteosarcoma were downloaded from the TARGET database (https://ocg.cancer.gov/programs/target) and the GEO database (http://www.ncbi.nlm.nih.gov/geo/). There were 14 samples (two normal human osteoblast cell lines and 12 tumor tissues) and 25 samples (two normal human osteoblast cell lines, four normal bones, and 19 osteosarcoma cell lines) in GSE12865 and GSE36001. In order to construct a prognostic risk model, cases from the TARGET or GEO database that are missing clinical characteristics were excluded from analysis. Finally, 93 of 101 patients with osteosarcoma and matched clinical information were extracted from TARGET as a training set. GSE21257 with 53 samples (53 tumor tissues) and matched clinical information was used as a validation set. The GSE21257 and GSE36001 samples were analyzed by the Illumina human-6 v2.0 expression BeadChip platform. The GSE12865 was analyzed by the Affymetrix Human Gene 1.0 ST array platform.

### Identification of Differentially Expressed Genes

Complete data on the TcoF-related gene symbol was downloaded from the Human Transcription Factor Database (http://bioinfo.life.hust.edu.cn/HumanTFDB#!/). At first, 1,025 TcoF-related genes were obtained. The GEO2R analysis for GSE36001 and GSE12865 was used to identify differentially expressed genes with a *p* value <0.05. Duplicate gene symbols from differentially expressed genes were deleted. The TcoF-related genes of differentially expressed genes were screened. Then, the genes were used for further analysis in the TARGET database and GSE21257.

### Construction and Validation of the Risk Score Model by Univariate Cox and LASSO Cox Regression Analyses

TcoF-related genes associated with prognosis were identified by the univariate Cox regression with a *p* value <0.2 to prevent omissions based on the TARGET database (Gokcal et al., 2021; Wang et al., 2021). After that, in order to screen candidate genes with an optimal number for this risk score model, the least absolute shrinkage and selection operator (LASSO) Cox regression with the “glmnet” R package was utilized for variable filters, and 10-fold cross-validation analysis was used for validation analysis.

Each osteosarcoma patient was given a risk score calculated by this model, and they were divided into two groups (low-risk and high-risk groups) by the median value of the risk score. The Kaplan–Meier (K-M curves) and receiver operating characteristic (ROC) curves were used to evaluate the robustness of the risk model. Principal component analysis (PCA) was analyzed by R language with the “stats” package. GSE21257 of the GEO database was regarded as the validation set to test the model. In addition, univariate and multivariate Cox analyses were performed to evaluate the prognostic efficiency of clinical features and our risk model.

### Correlation Analysis of the Risk Score With Immune Cells and Immune Signaling Pathways

With the progressive study of the immune microenvironment in tumors, multiple mechanisms of tumor immune escape have been discovered, and the effect of immune checkpoint inhibitors in tumors is exciting (Lei et al., 2020), indicating that the tumor immune microenvironment is the key to tumor immunotherapy. Thus, the immune infiltration of 16 immune cells and 13 immune-associated features in the TARGET and GSE21257 datasets were conducted by single-sample gene set enrichment analysis (ssGSEA). The R package “gsva” was used, and *p* < 0.05 was considered statistically significant.

### Construction of a Nomogram to Estimate the Clinical Outcome

The nomogram was constructed by the “rms” R package based on the TARGET and GSE21257. The association between the predicted outcome and the actual situation in 1, 2, and 3 years was tested by calibration curves. GSE21257 was used as a validation set to verify the nomogram.

### Relationship Between Genes in the Risk Model and Immune Cell Infiltration

The Tumor Immune Estimation Resource (TIMER) database (https://cistrome.shinyapps.io/timer/) can be used to reveal the association between the expression of genes and immune cell infiltration. The correlation analysis was performed to analyze the relationship between the genes in the risk model with immune cell infiltration (B cells, CD8^+^ T cells, CD4^+^ T cells, macrophages, neutrophils, and dendritic cells) using the data on sarcoma in this database.

### Functional Enrichment Analysis and Alteration of Five Genes in the Model

The cBioPortal dataset (https://www.cbioportal.org/) was used to study genetic variations. The genes in the risk model were based on the genomic data of osteosarcoma. GeneMANIA (http://www.genemania.org) contains genetic information, analysis of gene lists, and functional analysis of prioritized genes (Warde-Farley et al., 2010). Therefore, the model of gene interaction genes and their enrichment functions were carried out by this database.

### Gene Analysis of Prognosis in Patients With Sarcoma

The Kaplan–Meier plotter database (http://kmplot.com/analysis/) was able to assess the correlation between gene expression and survival from 21 tumor types. The influence of genes (*LMO2*, *MAML3*, *MTF2*, *RBPMS*, and *SIRT1*) on overall survival and disease-free survival in patients with sarcoma was analyzed.

### Cell Culture

Human osteosarcoma cell lines MNNG/HOS, 143B, MG63, and the human osteoblast cell line hFOB 1.19 were obtained from the American Type Culture Collection (ATCC, Manassas, VA, United States). The osteosarcoma cell line WELL5 was a kind gift from Dr. Wu Zhang of the Shanghai Institute of Hematology (Zhang et al., 2018). Four osteosarcoma cell lines were both cultured in Dulbecco modified Eagle medium (Gibco, 11965092) supplemented with 10% fetal bovine serum and 1% penicillin–streptomycin at 37°C with 5% CO_2_. hFOB 1.19 was cultured in DMEM/F12 medium (Gibco, 11320033) containing 10% FBS and 0.3 mg/ml G418 (Gibco, 10131035) at 34°C with 5% CO2.

To verify the expression of TcoF-related genes in osteosarcoma, the total RNA of four human osteosarcoma cell lines (MNNG/HOS, 143B, MG63, and WELL5) and one human osteoblast cell line (hFOB 1.19) were isolated by using the RNAsimple Total RNA Kit (TIANGEN, China). cDNA was obtained with HyperScriot III First-Strand cDNA Synthesis Kit (NovaBio, China) after removing gDNA. Finally, qRT-PCR was performed by LightCycler480 II, and GAPDH was used as an internal control gene. Validation of each gene was performed three times as biological replications, and the results of osteosarcoma cell lines and the osteoblast cell line were analyzed by Student’s t test. The primer sequences of TcoF-related genes are listed as follows:

LMO2: TTG​CAA​CAT​CCT​CAA​AGA​ACA​GC (forward);

LMO2: GAG​GGC​AGG​TGG​GGG​TAT​TT (reverse);

MAML3: CAA​TCA​GTT​TCA​AGG​TTC​TCC​C (forward);

MAML3: GACGTTCGCATGCTCTGG (reverse);

MTF2: CCT​GTA​TGA​AGC​GGT​TGG​C (forward);

MTF2: TTT​ATG​TCC​ATC​CTG​CCC​CT (reverse);

RBPMS: TTC​ACT​GCA​TGC​CCA​GAT​GC (forward);

RBPMS: CAC​CTG​GGA​CAT​AGT​ATT​CAG​C (reverse);

SIRT1: AAG​GCC​ACG​GAT​AGG​TGT​CT (forward);

SIRT1: TGG​GAA​GTC​TAC​AGC​AAG​GC (reverse).

### Immunohistochemistry Analysis

The study protocol was part of our longitudinal observational clinical research project registered at clinicaltrial.gov (trial ID: NCT03108677) and approved by the Ethics Review Committee of Shanghai Ruijin Hospital. To make the results more convincing, immunohistochemistry analysis was performed using tissue microarray (TMA), which is an effective high-throughput technique platform for the study of tumor molecular pathology. TMA was constructed by 110 patients who have been diagnosed with osteosarcoma in Shanghai Ruijin Hospital during 2013–2019. All the tumor tissue samples from the patients were histologically confirmed as osteosarcoma and reviewed independently by two pathologists at Ruijin Hospital. For immunochemistry analysis, the TMA of osteosarcoma tissues and 11 prepared paraffin blocks of normal osseous tissues were sliced at 4 μm thickness. Then, the sections were deparaffinized and rehydrated with xylene and ethanol. After heat-induced antigen retrieval, endogenous peroxidase activity blockage, and serum sealing, the samples were incubated with rabbit anti-MTF2 (Proteintech, 16208-1-AP, 1:50) and anti-RBPMS (Abcam, ab152101, 1:100) antibodies at 4°C overnight. Slices were covered with the horseradish peroxidase-coupled goat anti-rabbit secondary antibody (CST, 7074, 1:200) at room temperature for 50 min and stained with diaminobenzidine. The nuclei were counterstained by hematoxylin. Finally, the samples were dehydrated and mounted. All slices were visualized by using the microscope (Nikon DS-U3).

We graded the intensity of staining as follows: 0, no staining; 1, yellow; 2, pale brown; and 3, dark brown and scored the extent of staining based on the rate of the positive cell (0, <5%; 1, 5–25%; 2, 26–50%; 3, 51–75%; 4, 76–100%). By multiplying the color value with the positive cell rate, we obtained the final IHC score as follows: 0–2 (–), 3–4 (+), 5–8 (++), and 9–12 (+++).

### Statistical Analysis

R language software (version 4.0.5) and Perl software (version 5.28) were used for data analysis. Excel 2019 software was used to organize the data. *p* < 0.05 was set as significance.

## Results

### Overall Design of the Study

**FIGURE 1:**
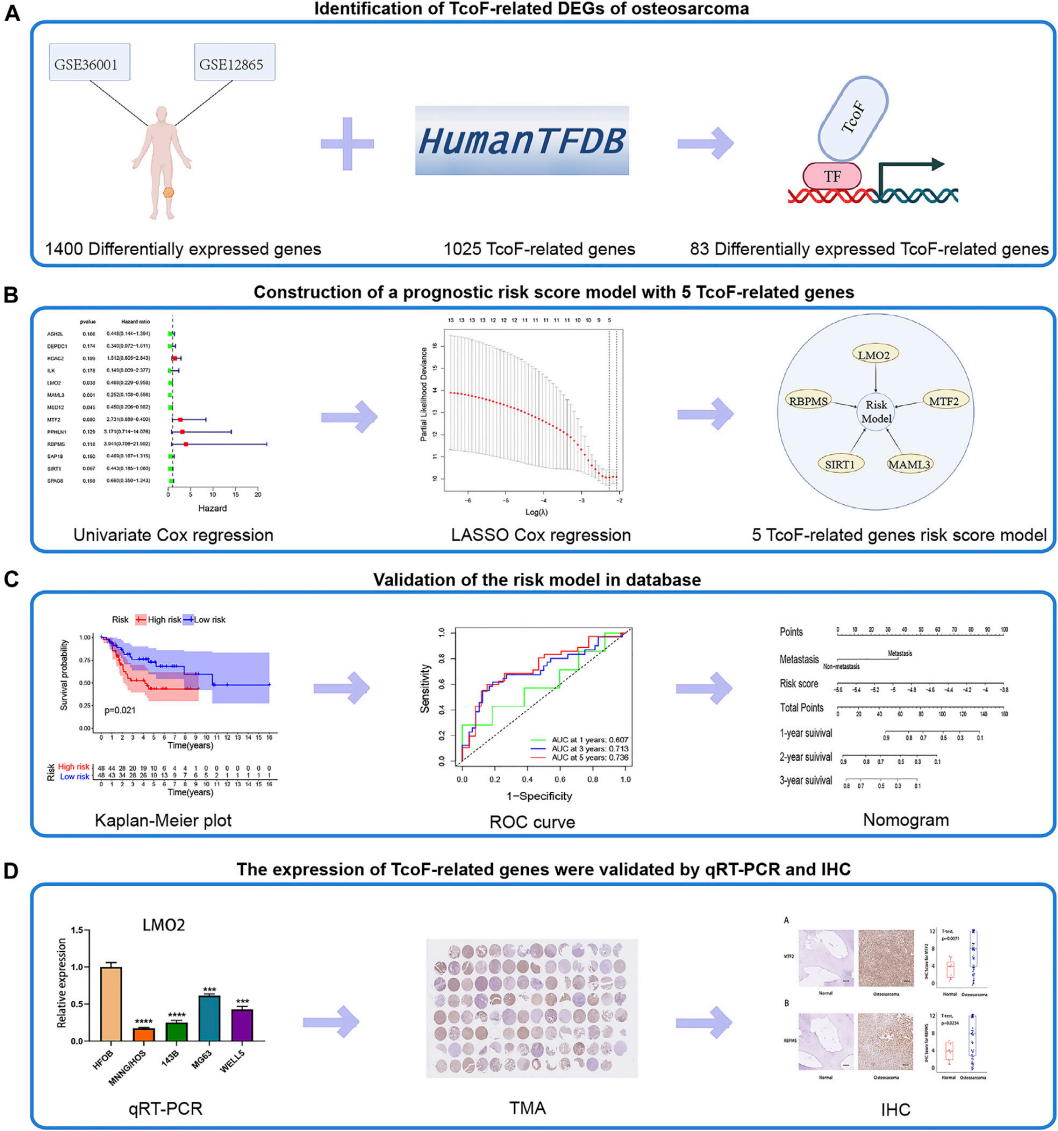
Flow chart of the study. **(A)** 83 TcoF-related DEGs of osteosarcoma were identified through two GEO databases (GSE36001 and GSE12865) and the Human Transcription Factor database. **(B)** Prognostic risk score model with five TcoF-related genes was constructed based on the TARGET database. **(C)** Validation of the risk model in the database. **(D)** Expressions of TcoF-related genes were validated by qRT-PCR and IHC. TcoF, transcription co-factor; DEG, differentially expressed gene; GEO, Gene Expression Omnibus; TARGET, Therapeutically Applicable Research to Generate Effective Treatments database; qRT-PCR, quantitative real-time PCR; TMA, tissue microarray; IHC, immunohistochemistry.

The flow chart for this study is shown in Figure 1. First, 1,400 differentially expressed genes were screened from GSE36001 (two normal human osteoblast cell lines, four normal bones, and 19 osteosarcoma cell lines) and GSE12865 (two normal human osteoblast cell lines and 12 tumor tissues). According to the Human Transcription Factor database and differential expression analysis, 83 differentially expressed TcoF-related genes in patients with osteosarcoma were screened (Figure 1A). Second, 13 genes were selected by univariate Cox regression. Five TcoF-related genes were screened out of 13 candidate genes using LASSO Cox regression analysis with the smallest bias, and they were combined to construct a prognostic risk model (Figure 1B). Third, the risk model was tested by K-M and ROC curve. Then, combining with the risk score and clinical factors, the nomogram of osteosarcoma was constructed and was evaluated by calibration and C-index (Figure 1C). Finally, the expressions of TcoF-related genes were validated in different cell lines by qRT-PCR and in different tissue samples by IHC (Figure 1D).

### Differential Gene Analysis

**FIGURE 2:**
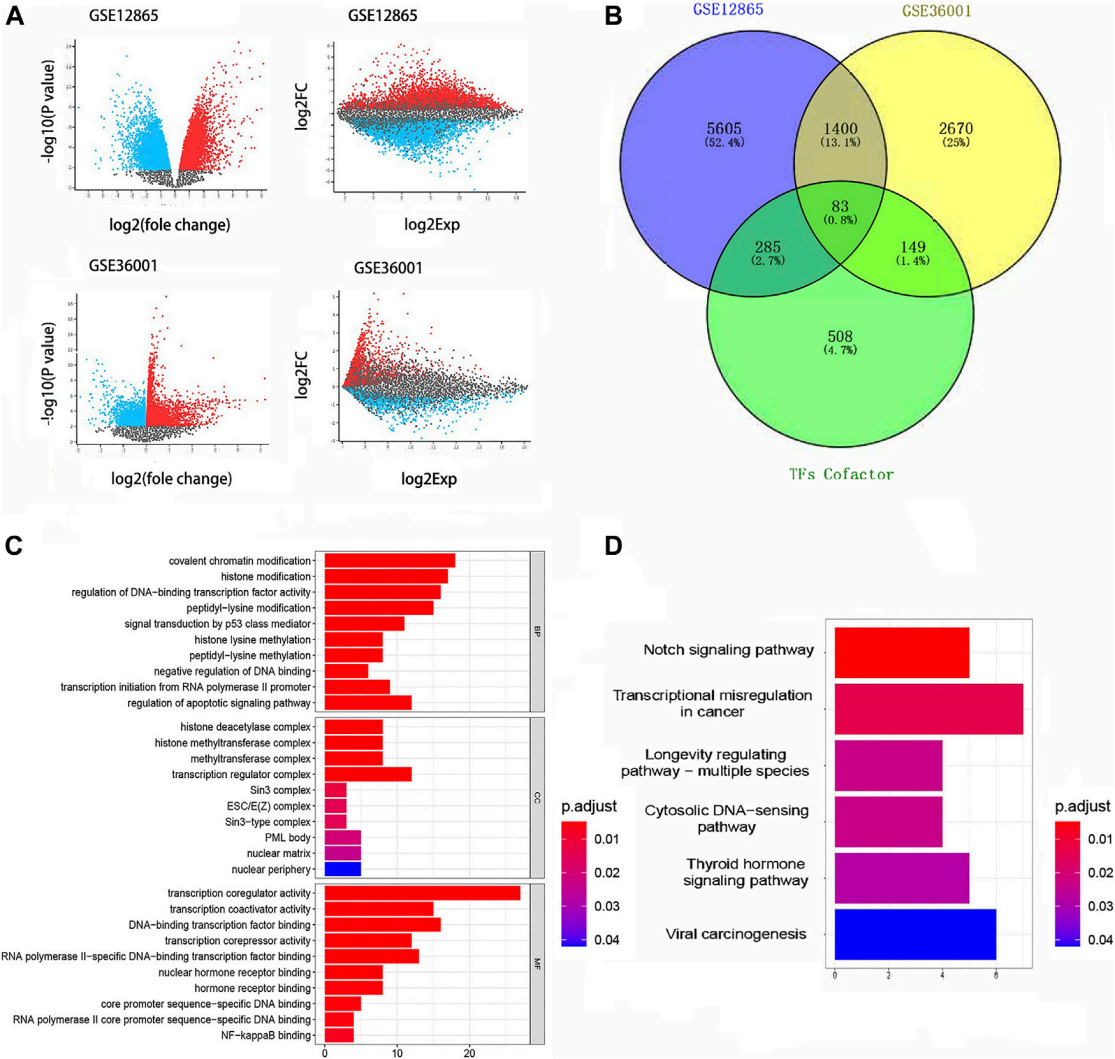
Results of differential gene analysis. **(A)** Differentially expressed genes in GSE36001 and GSE12865. **(B)** 83 differentially expressed TcoF-related genes were screened from differentially expressed genes. **(C)** GO bar graph for genes in BP, CC, and MF. **(D)** Result of the KEGG pathway enrichment analysis. GO, Gene Ontology; KEGG, Kyoto Encyclopedia of Genes and Genomes.

Differentially expressed genes in GSE36001 and GSE12865 are shown in Figure 2A, of which 83 differentially expressed TcoF-related genes were screened (Figure 2B). Gene Ontology (GO) analysis showed 83 genes were mainly concentrated on covalent chromatin modification in the biological process (BP), transcription regulator complex in cellular component (CC), and transcription coregulator activity in molecular function (MF) (Figure 2C). The result of the Kyoto Encyclopedia of Genes and Genomes (KEGG) pathway enrichment analysis is shown in Figure 2D.

### Construction and Validation of the Risk Score Model by Univariate Cox and LASSO Cox Regression Analyses

**FIGURE 3:**
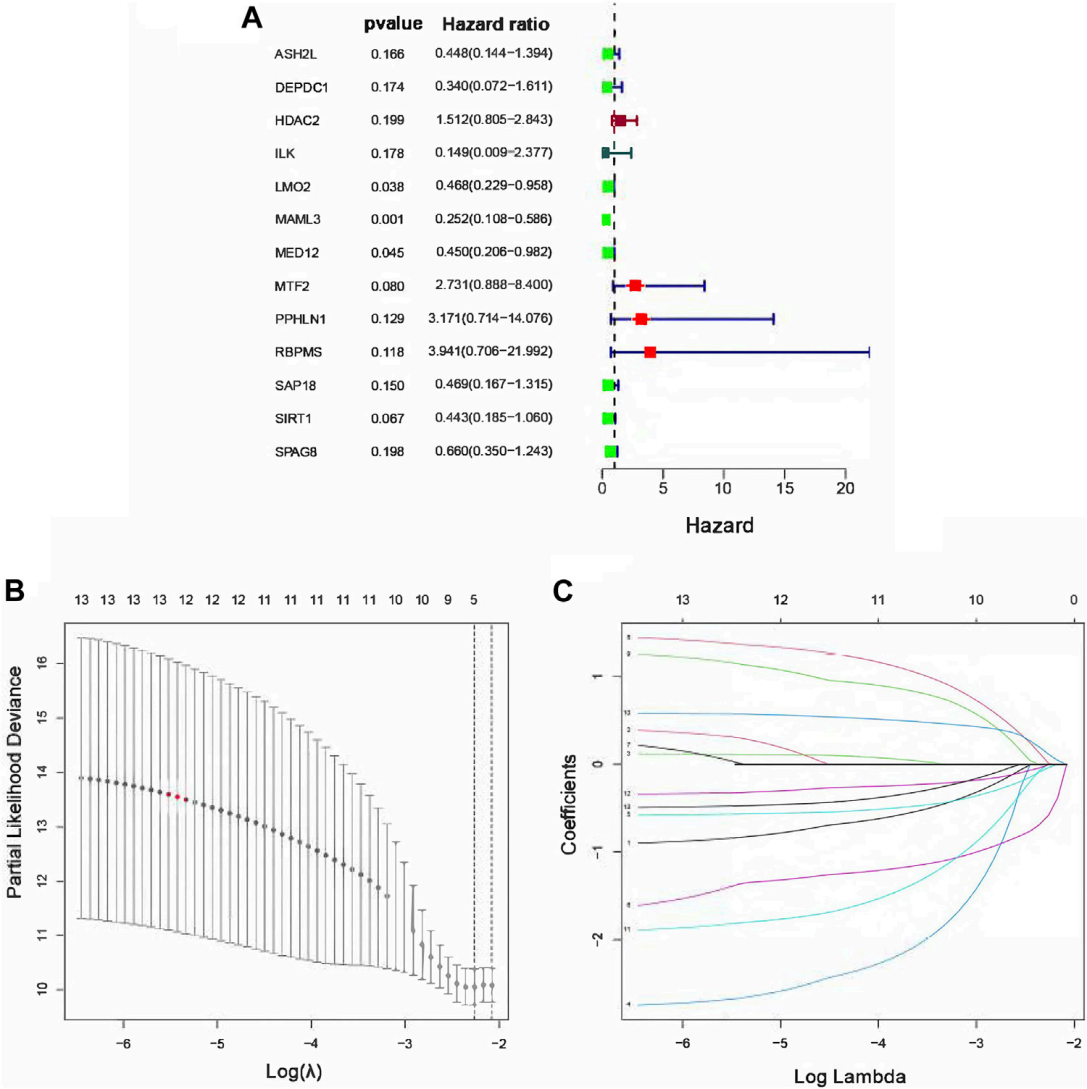
Construction of the risk score model for osteosarcoma patients. **(A)** Hazard ratio of univariate Cox analysis for TcoF-related DEGs. **(B)** Distribution of LASSO coefficients for five genes. **(C)** Coefficients for seven genes analyzed by LASSO. DEGs, differentially expressed genes.

In order to identify the TcoF-related genes associated with prognosis in osteosarcoma, 83 differentially expressed TcoF-related genes mentioned earlier were analyzed by the univariate Cox regression, and 13 TcoF-related candidate genes were selected with a *p* value <0.2 based on the TARGET database (Figure 3A). Then, we performed LASSO Cox regression to screen a relatively small group of 13 TcoF-related candidate genes with a nonzero regression coefficient and further identify five genes (*LMO2*, *MAML3*, *MTF2*, *RBPMS*, and *SIRT1*) which were finally used to construct the prognostic risk model (Figure 3B, C).

### TcoF-Related Gene Prognostic Risk Score Model

A risk model with five TcoF-related genes was constructed by the aforementioned methods, and the formula of the risk score was as follows:

riskScore=(the expression of LMO2∗−0.0376) + (the expression of MAML3∗ −0.5804) + (the expression of MTF2∗ 0.0136)+(the expression of RBPMS∗ 0.1161)+(the expression of SIRT1∗ −0.0061).

**FIGURE 4:**
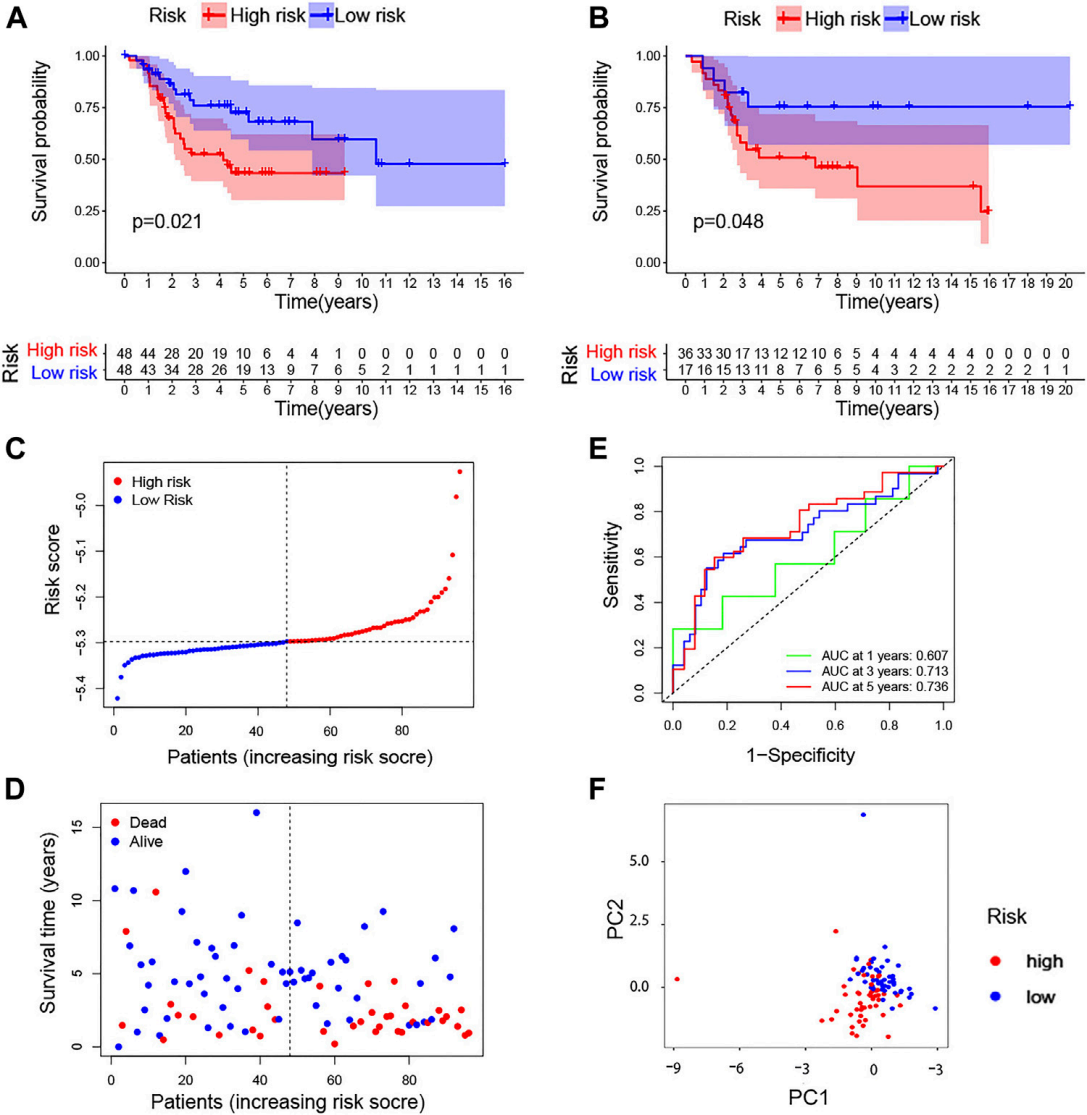
TcoF-related gene prognostic risk score model. **(A,B)** Survival analysis to verify the prognostic model based on TARGET and GSE21257. **(C)** Distribution of risk scores of each osteosarcoma patient. **(D)** Osteosarcoma patients’ survival time according to the risk score model. **(E)** Time-dependent ROC curves for osteosarcoma patients based on TARGET. **(F)** PCA plot for osteosarcoma patients based on the risk score based on TARGET. PCA, principal component analysis.

K-M curves showed significant differences in the survival rate in both TARGET (*p* = 0.021) and GSE21257 (*p* = 0.048) (Figure 4A, B). According to the median value of the formula results, the risk score and survival time of each osteosarcoma patient based on TARGET are shown in Figure 4C, D. Moreover, the time-dependent ROC curves based on TARGET demonstrated that this risk model was convincible (1-year AUC: 0.607; 3-year AUC: 0.713; 5-year AUC: 0.736) (Figure 4E). The PCA also showed that the high- and low-risk groups were obviously distinguished into two parts (Figure 4F). We again used data from the TARGET database to construct a risk model using the 13 genes obtained from a univariate Cox regression analysis and plotted survival curves. Although it can distinguish between high-risk and low-risk groups of patients well, when we used GSE21257 to test the ability of the model to predict risk again with these 13 genes, its power was very low with *p* = 0.305 (Supplementary Figure S6).

**FIGURE 5:**
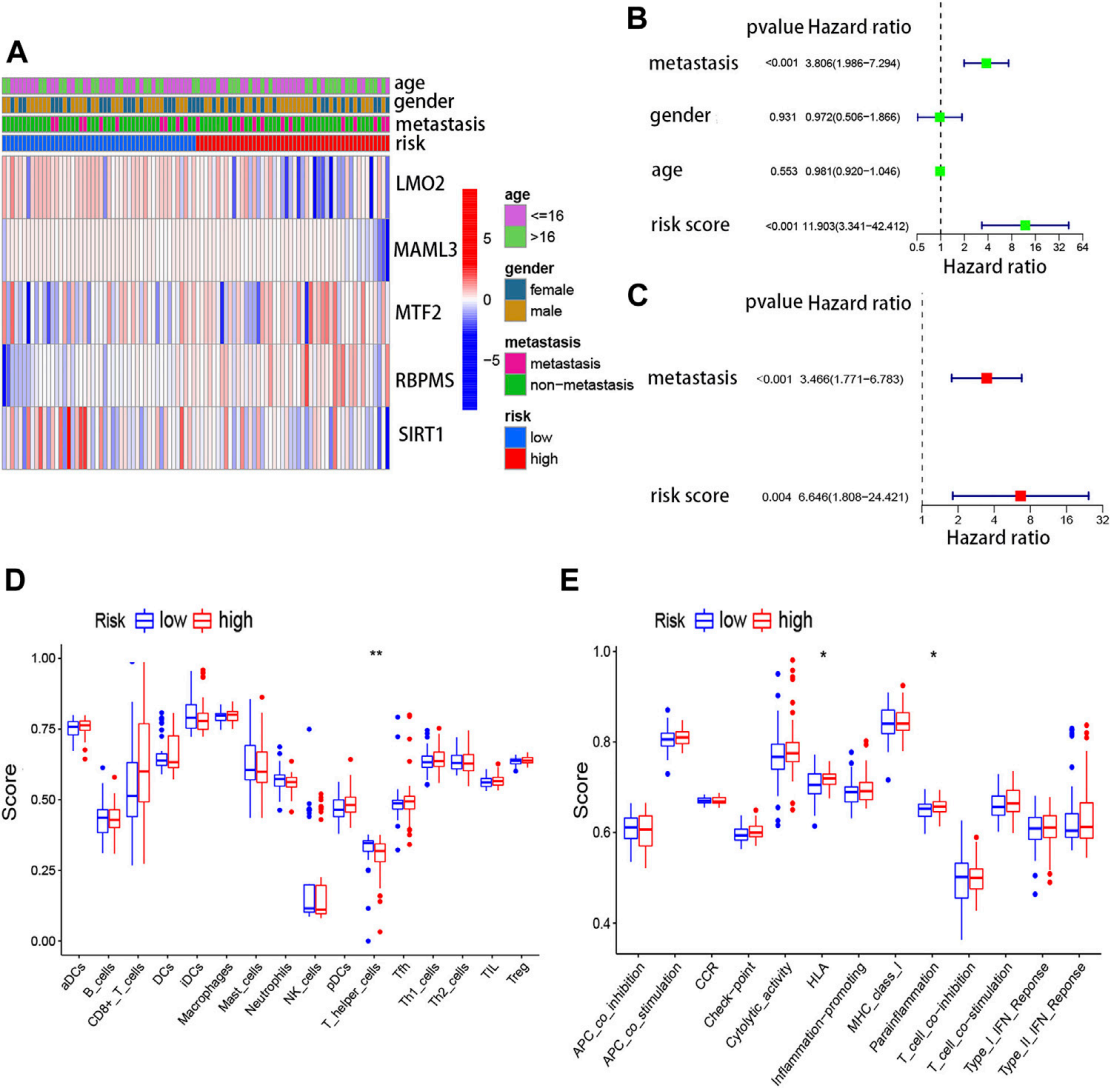
Risk score model and immune cells, as well as immune signaling pathways. **(A)** Expressions of five genes in the risk model between high- and low-risk scores. **(B)** Univariate Cox regression analysis for the risk score based on TARGET. **(C)** Multivariate Cox regression analysis for the risk score based on TARGET. **(D)** Association between the risk score and different immune cells based on TARGET and GSE21257. **(E)** Association between the risk score and different immune features based on TARGET and GSE21257.

In addition, the expression of five genes in the risk model between high- and low-risk scores is shown in Figure 5A. Meanwhile, to evaluate whether clinical features (metastatic, gender, and age) and risk score could be the independent prognostic indicators, univariate and multivariate Cox analyses were performed based on the TARGET database. Through univariate regression analysis, we determined that metastasis and risk score were associated with osteosarcoma prognosis (*p* value <0.05). The hazard ratios (HR) for metastasis and risk score were 3.806 and 11.903, respectively (Figure 5B). Then, we further confirmed that metastasis and risk score were independent prognostic indicators for osteosarcoma using multivariate regression analysis (*p* value <0.05). The hazard ratios (HR) for metastasis and risk score were 3.466 and 6.646, respectively (Figure 5C). In short, this model was reliable to predict the prognosis of osteosarcoma.

### Correlation Between the Risk Score and Immune Cells and Immune Signaling Pathways

To further understand the association between five TcoF-related genes’ prognostic risk model and immune infiltration, ssGSEA was carried out. Two immune cells (CD8^+^ T cells and T helper cells) and two immune-related pathways were significantly (HLA and para inflammation) related to the risk score in TARGET (Figure 5D, E). To some extent, this suggested that the immune microenvironment may relate to the occurrence of osteosarcoma.

### Construction and Validation of the Nomogram

**FIGURE 6:**
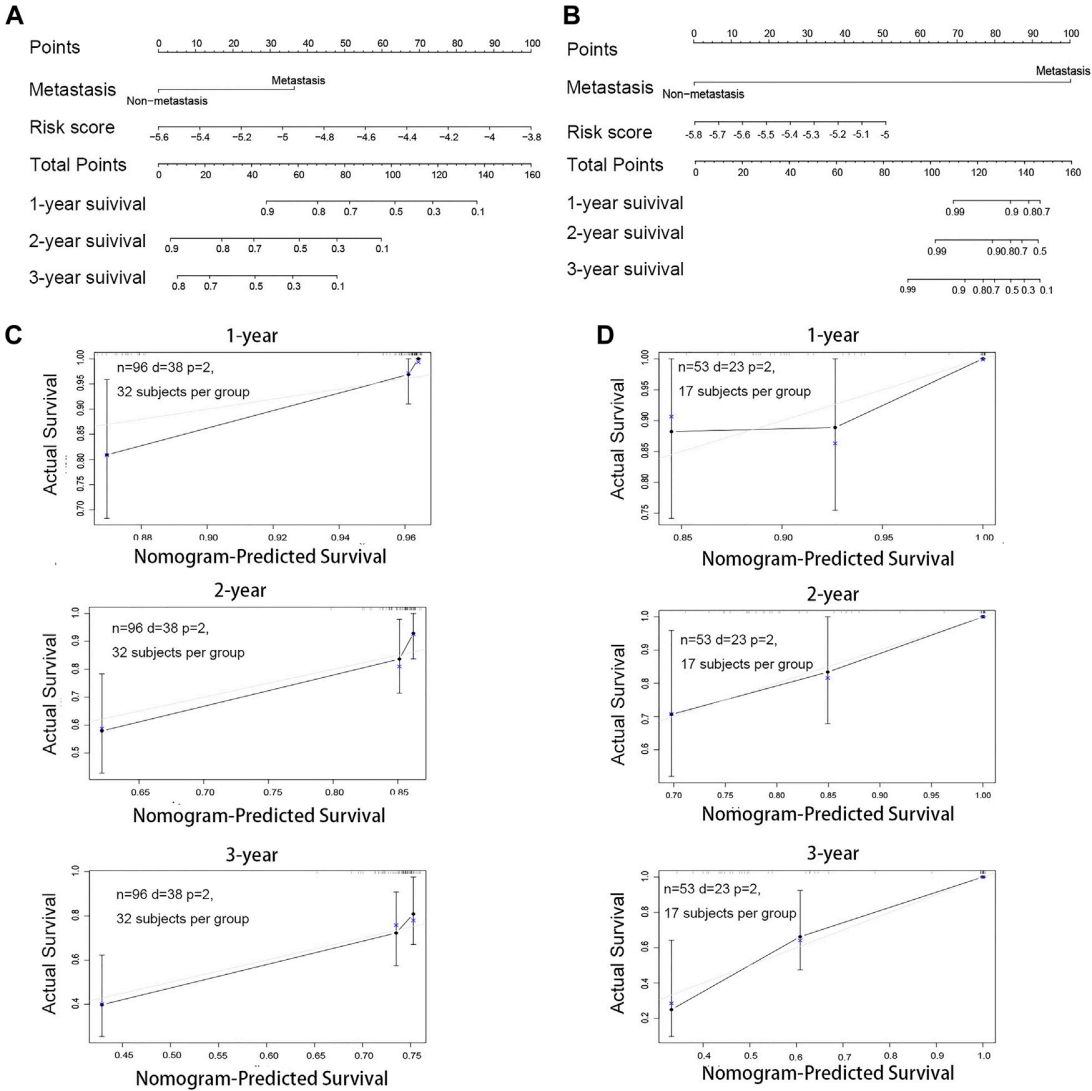
Construction and validation of the nomogram for osteosarcoma patients. **(A)** Nomogram for predicting the 1-, 2-, and 3-year survival rate by metastasis and the risk score based on TARGET. **(B)** Nomogram for predicting the 1-, 2- and 3-year survival rate by metastasis and the risk score based on GSE21257. **(C)** Calibration curve of the 1-, 2- and 3-year survival rate based on TARGET. **(D)** Calibration curve of the 1-, 2-, and 3-year survival rate based on GSE21257.

Next, we used a nomogram to predict the prognosis of patients by integrating the risk factors of metastasis and risk score. The nomogram for osteosarcoma patients’ survival was established to forecast the survival probabilities of 1-, 2-, and 3-year overall survival time based on TARGET (Figure 6A) and GSE21257 (Figure 6B). To investigate whether the actual survival time was in line with the predicted survival rate in 1, 2, and 3 years, the calibration curves were plotted. The C-index of this nomogram in predicting overall survival time was 0.738 in TARGET (Figure 6C) and 0.804 in GSE21257 (Figure 6D). The calibration curve and C-index both indicated the high reliability of the nomogram.

### Relationship Between Genes in the Risk Model and Immune Cell Infiltration

In order to reveal the association between the expressions of five TcoF-related genes (*LMO2*, *MAML3*, *MTF2*, *RBPMS*, and *SIRT1*) in the risk model and immune cell infiltration, the data on sarcoma from the TIMER database were collected. The results showed that CD4^+^ T cells, macrophages, neutrophils, and dendritic cells all had a significant correlation with the gene of LMO2. Macrophages, neutrophils, and dendritic cells were also related to the gene of MAML3. CD4^+^ T cells, macrophages, and dendritic cells were related to the gene of MTF2. CD4^+^ T cells and dendritic cells were related to the gene of SIRT1 (Supplementary Figure S1). The results of correlation analysis showed that the risk model had the predictive power for potential immune cell infiltration.

### Functional Enrichment Analysis and Alteration of Five Genes in the Model

In addition, the cBioPortal dataset was used to reveal genetic variations of the five TcoF-related genes. Mutations of each gene (*LMO2*, *MAML3*, *MTF2*, *RBPMS*, and *SIRT1*) in sarcoma were 6, 7, 6, 3, and 5%, respectively, with the most common changes in the mRNA expression. However, mutations in each gene were more limited. The combination of the five genes enabled the model to comprehensively reflect the patient’s condition (Supplementary Figure S2A, B). Furthermore, the model of gene interaction genes and their enrichment function was built by GeneMANIA. The network of these five genes was associated with many functions, including activation of transcription factor binding and modulation of DNA-templated transcription initiation (Supplementary Figure S2C). The results further confirmed these five genes in the risk model were TcoF-related genes. Therefore, the five TcoF-related genes’ prognostic risk score model was convincing.

### Gene Analysis of Prognosis in Patients With Sarcoma

The correlation between five TcoF-related genes’ expression and survival was assessed by using the Kaplan–Meier plotter database. The Kaplan–Meier plotter database is known as an authoritative database that can assess the correlation between the gene expression and survival in various tumor types (Lanczky & Gyorffy, 2021). Sources of the database include GEO, EGA (European Genome-Phenome Archive), and TCGA (The Cancer Genome Atlas). The influence of five genes (*LMO2*, *MAML3*, *MTF2*, *RBPMS*, and *SIRT1*) on overall survival and disease-free survival in patients with sarcoma is shown in Supplementary Figure S3A-E and Supplementary Figure S4A-E. A high expression of the *MTF2* gene and low expressions of *LMO2*, *MAML3*, and *RBPMS* genes were associated with poor prognosis, while *LMO2* and *MAML3* were related to disease-free survival in patients.

### Validation of the TcoF-Related Genes in Osteosarcoma and Osteoblast Cell Lines

**FIGURE 7:**
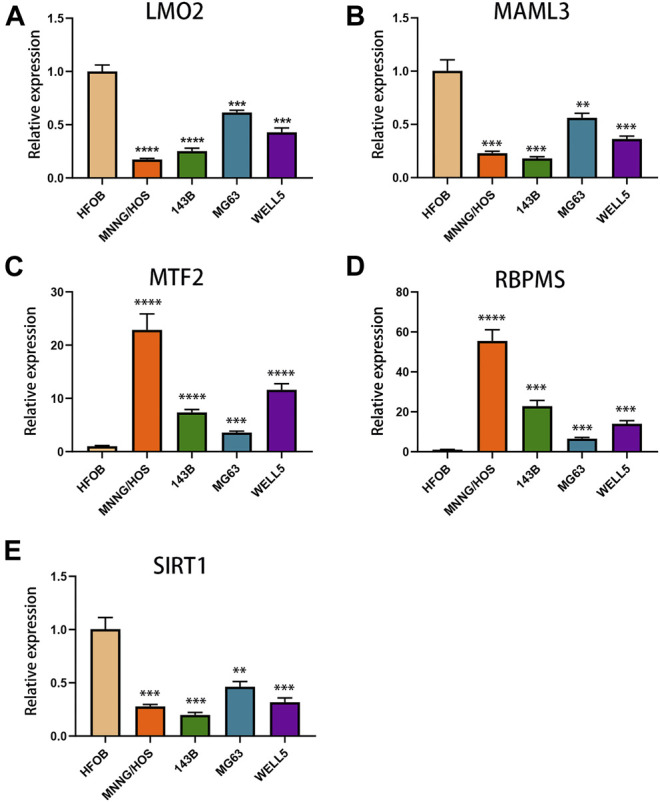
Expressions of TcoF-related genes in osteosarcoma and osteoblast cell lines. **(A)** Expression of LMO2 in osteosarcoma and osteoblast cell lines. **(B)** Expression of MAML3 in osteosarcoma and osteoblast cell lines. **(C)** Expression of MTF2 in osteosarcoma and osteoblast cell lines. **(D)** Expression of RBPMS in osteosarcoma and osteoblast cell lines. **(E)** Expression of SIRT1 in osteosarcoma and osteoblast cell lines. **p < 0.01; ***p < 0.001; ****p < 0.0001.

The expression levels of TcoF-related genes in human osteosarcoma and osteoblast cell lines were detected through qRT-PCR analyses. The results showed that the expressions of MTF2 and RBPMS were upregulated in all osteosarcoma cell lines (MNNG/HOS, 143B, MG63, and WELL5) compared with the osteoblast cell line (Figure 7C, D). In contrast, the expressions of *LMO2*, *MAML3*, and *SIRT1* were downregulated in all osteosarcoma cell lines (Figure 7A, B, E).

### Validation of the TcoF-Related Genes in Osteosarcoma Tissues and Normal Osseous Tissues

Finally, tissue microarrays (TMAs) of 110 osteosarcoma patients and 11 normal osseous controls were selected for IHC analysis. The detailed clinical information of patients is shown in Table 1. The expressions of *MTF2* and *RBPMS* in osteosarcoma tissues and normal osseous tissues were analyzed by IHC. MTF2 and RBPMS were both highly expressed in osteosarcoma tissues compared with normal osseous controls with *p* values of 0.0071 and 0.0234, respectively.

**TABLE 1:** Clinical information for patients with osteosarcoma.

Characteristic	Osteosarcoma	Normal
Gender		
Female	52 (47.3%)	6 (54.5%)
Male	58 (52.7%)	5 (45.5%)
Age		
<20	60 (54.5%)	6 (54.5%)
≥20	50 (45.5%)	5 (45.5%)
Location		
Thigh	65 (59.1%)	7 (63.6%)
Calf	18 (16.4%)	4 (36.4%)
Upper arm	16 (14.5%)	—
Pelvis	8 (7.3%)	—
Forearm	2 (1.8%)	—
Scapula	1 (0.9%)	—

(Figures 8A, B). The overall results of the tissue microarray for 110 osteosarcoma tissues are placed in Supplementary Figure S5.

**FIGURE 8:**
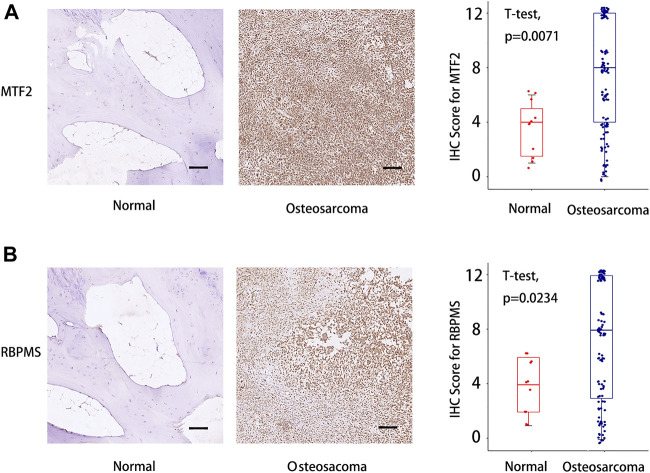
IHC score difference analysis for MTF2 and RBPMS in osteosarcoma patients. **(A)** IHC score analysis for MTF2. **(B)** IHC score analysis for RBPMS. Scale bar = 100 um. IHC, immunohistochemistry. Supplementary Figure S1 The association between the expression of five TcoF-related genes (*LMO2*, *MAML3*, *MTF2*, *RBPMS*, and *SIRT1*) in the risk model and immune cell infiltration.

## Discussion

Osteosarcoma is one of the most common primary malignant bone tumors with poor prognosis, which mainly occurs in children and adolescents (Chow et al., 2021; Rojas et al., 2021). Although clinical therapy is constantly updated, there is still a lack of models for predicting tumor prognosis, which makes it difficult to make optimal clinical decisions. In this study, we constructed a prognostic risk model with five TcoF-related genes and validated the applicability and robustness with K-M and ROC curves. The risk model showed a significant association with immune cells and immune-related functions. In addition, the nomogram constructed with a risk score and clinical factors further supported the reliability of this model. Finally, we selected MTF2 and RBPMS for IHC validation in osteosarcoma tissues and normal osseous tissues. To the best of our knowledge, this was the first study to use TcoF-related genes to construct a risk model to predict the prognosis of osteosarcoma patients.

TcoF is one type of protein that performs the function of transcriptional regulation by interacting with transcription factors (TFs) (Latchman, 1997; Xu et al., 1999; Gill, 2001). Accumulating results have shown that TcoFs play critical roles in signal transmission, TF-DNA binding modulation, and chromatin modification (Martinez, 2002; Thomas and Chiang, 2006). However, the dysfunction of TcoFs has a well-established association with many diseases. In recent years, the relationship between TcoFs and tumors has been widely revealed. Raffaella et al. showed that ZNF521 (the stem cell-associated TcoF) was involved in the proliferation, growth as spheroids, and ability to generate clones of medulloblastoma (MB) cells (Spina et al., 2013). ZNF521 also contributed to pre-B-cell lymphomagenesis through modulation of the pre-B-cell receptor signaling pathway (Hiratsuka et al., 2016). SATB2 (special AT-rich binding protein - 2), as a type of TcoF, could induce epithelial–mesenchymal transition and metastasis and be used as a diagnostic biomarker for cancer (Roy et al., 2020). JMY (junction-mediating and regulatory protein) was a TcoF that played a significant role in regulating the tumor suppressor p53, which initiated a checkpoint response culminating in cell cycle arrest or apoptosis (Coutts et al., 2010). Therefore, TcoFs play essential roles in the development of tumors. We reasonably hypothesized that TcoFs may be correlated with the diagnosis and prognosis of osteosarcoma.

To verify our speculation, we screened several publicly available databases and discovered five TcoF-related genes (*LMO2*, *MAML3*, *MTF2*, *RBPMS*, and *SIRT1*). Constructed by these five TcoF-related genes, our risk model could classify osteosarcoma patients into high- and low-risk scores and further predict their survival rates of 1-, 2-, and 3-year overall survival time combined with their clinical features. Notably, some of the selected genes have been reported to be associated with the occurrence, development, and prognosis of tumors. The expression of LMO2 has been identified as one of the best prognostic markers for prolonged survival in patients with diffuse large B-cell lymphoma, following immunochemotherapy (Parvin et al., 2019). It was also found to be involved in the invasion and metastasis of prostate cancer (Ma et al., 2007). Overexpression of MAML3 can increase tumorigenicity and even induce a malignant phenotype under hypoxia (Onishi et al., 2016; Alzofon et al., 2021). In addition, MTF2 deficiency could predict refractory acute myeloid leukemia at initial diagnosis (Maganti et al., 2018). These further provide evidence of the feasibility of the TcoF-related genes we selected for the clinical prognosis risk model.

In fact, increasing risk models based on DEGs were performed to predict the prognosis of a variety of diseases, which can also be found in the research of the osteosarcoma field (Fu et al., 2021; Li et al., 2021). Instead of building risk models using DEGs directly, we further selected TcoF-related genes among DEGs due to the unexplored roles of TcoF-related genes in prognosis. We proved that the five TcoF-related genes’ risk model makes an inspiring effect in predicting the diagnosis of osteosarcoma, which is a creative discovery. In addition, the procedure of univariate Cox regression and LASSO Cox regression for selecting the TcoF-related DEGs can minimize the error between the predicted and the real survival time, which is not available for the risk models constructed by Li et al. The good robustness of these five TcoF-related genes' risk model revealed that TcoFs may act as independent factors to predict the prognosis of osteosarcoma.

The limitations to the study include the lack of deep investigation of the underlying mechanism of selected TcoFs in the pathogenesis of osteosarcoma. Given the fundamental purpose of this study is to construct ideal prediction models for the prognosis of osteosarcoma, studies on specific molecular mechanisms of selected TcoFs in osteosarcoma are our future research directions. Another limitation is that more data on samples from osteosarcoma patients are acquired for further validation due to the scarce databases for osteosarcoma.

## Conclusion

In a word, the study revealed significant differential expressions of TcoF-related genes in different cell lines and tissue samples. The risk score provided by the model was the independent factor for the prognosis of osteosarcoma patients. Therefore, the constructed five TcoF-related genes’ risk model was able to predict the prognosis and may provide potentially effective novel therapeutic targets for osteosarcoma patients.

## Data Availability

The datasets presented in this study can be found in online repositories. The names of the repository/repositories and accession number(s) can be found in the article/Supplementary Material.
