# Supplementary figures and images for: Identification of the Transcription Co-Factor–Related Gene Signature and Risk Score Model for Osteosarcoma
Source: Front Genet. 2022 Jun 6;13:862803. doi: 10.3389/fgene.2022.862803 (PMC9207420; doi:10.3389/fgene.2022.862803)

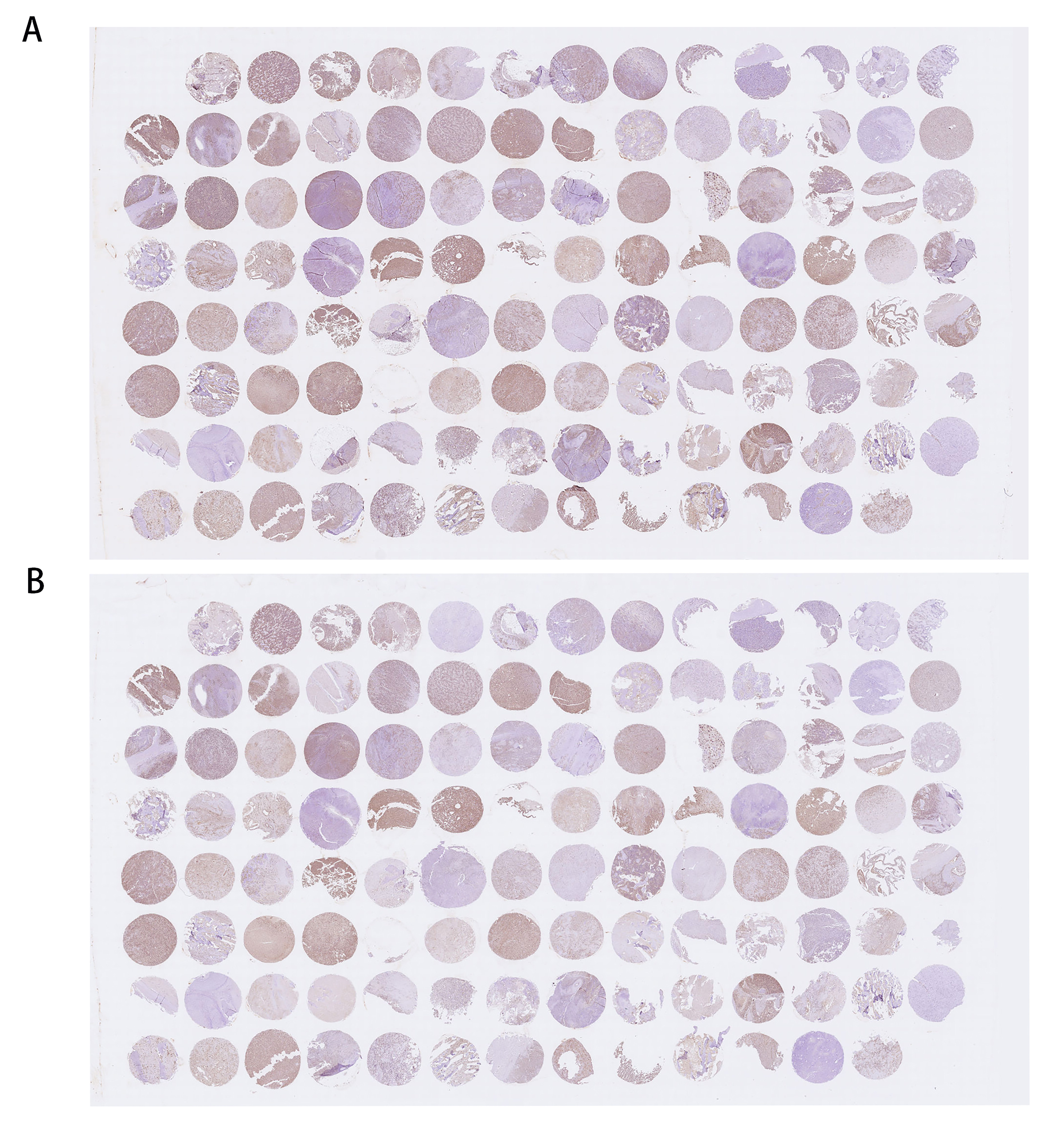

Supplement: Supplementary file 1 [file Image5.jpg]

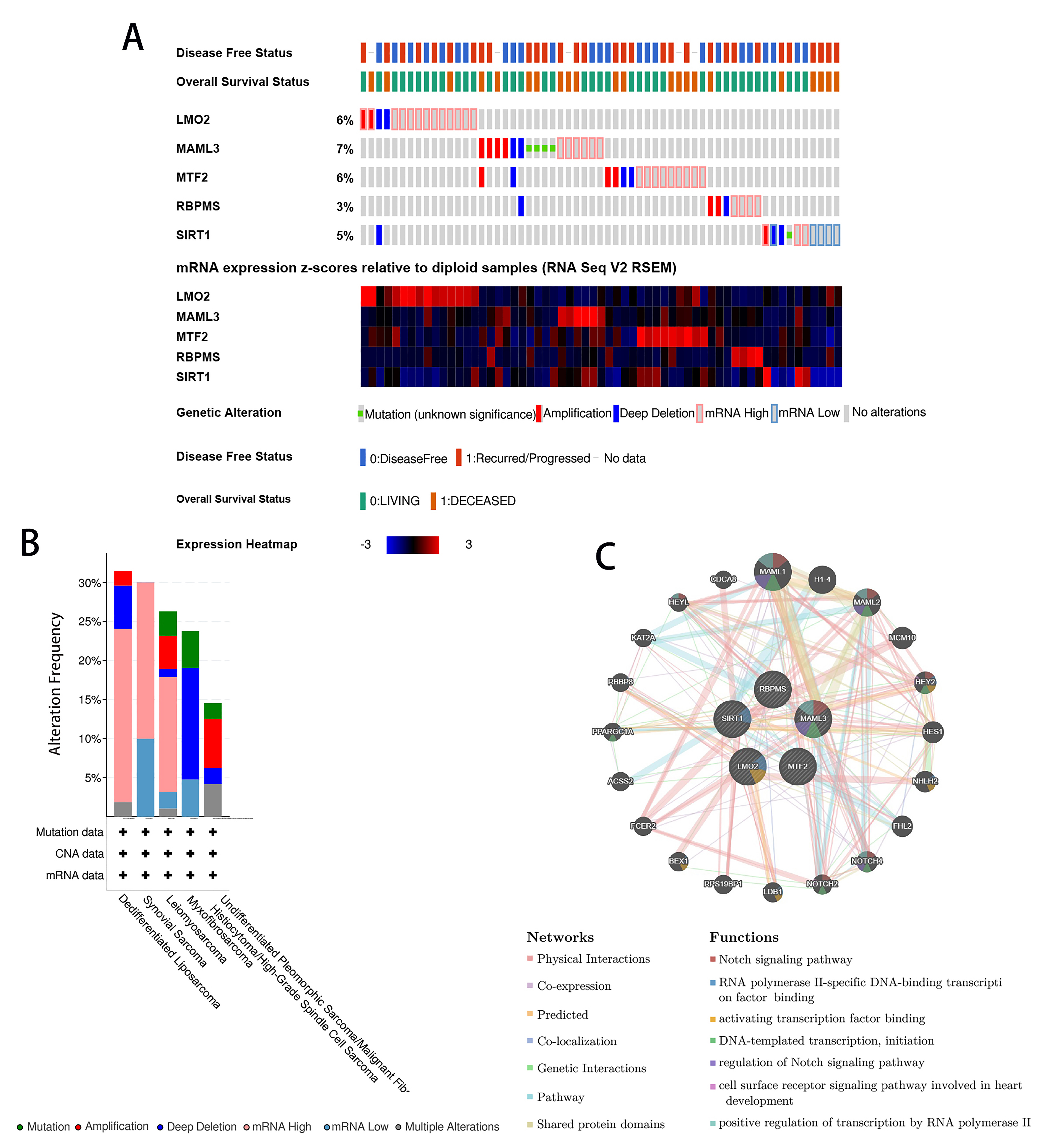

Supplement: Supplementary file 4 [file Image2.jpg]
