# Supplementary material for: Identification of the Transcription Co-Factor–Related Gene Signature and Risk Score Model for Osteosarcoma
Source: Front Genet. 2022 Jun 6;13:862803. doi: 10.3389/fgene.2022.862803 (PMC9207420; doi:10.3389/fgene.2022.862803)
Supplement: Supplementary file 7 [file DataSheet1.docx]

**Supplementary Information for**

**Identification of Transcription Co-factor Related Gene Signature and Risk Score Model for Osteosarcoma**

Zhijian Jin^1†^, Jintao Wu^1†^, Jianwei Lin^2†^, Jun Wang^1^, Yuhui Shen^1*^

^1^Department of Orthopaedics, Ruijin Hospital, Shanghai Jiao Tong University School of Medicine. Shanghai, Shanghai, China

^2^Department of General Surgery, Ruijin Hospital, Shanghai Jiao Tong University School of Medicine. Shanghai, Shanghai, China

*** Correspondence:**

Yuhui Shen [yuhuiss@163.com](mailto:yuhuiss@163.com)

This file includes:

Figures S1-S5

**Supplementary Figure S1**





**Supplementary Figure S1.** The association between the expression of 5 TcoF-related genes (LMO2, MAML3, MTF2, RBPMS, SIRT1) in risk model and immune cell infiltration.

**Supplementary Figure S2**


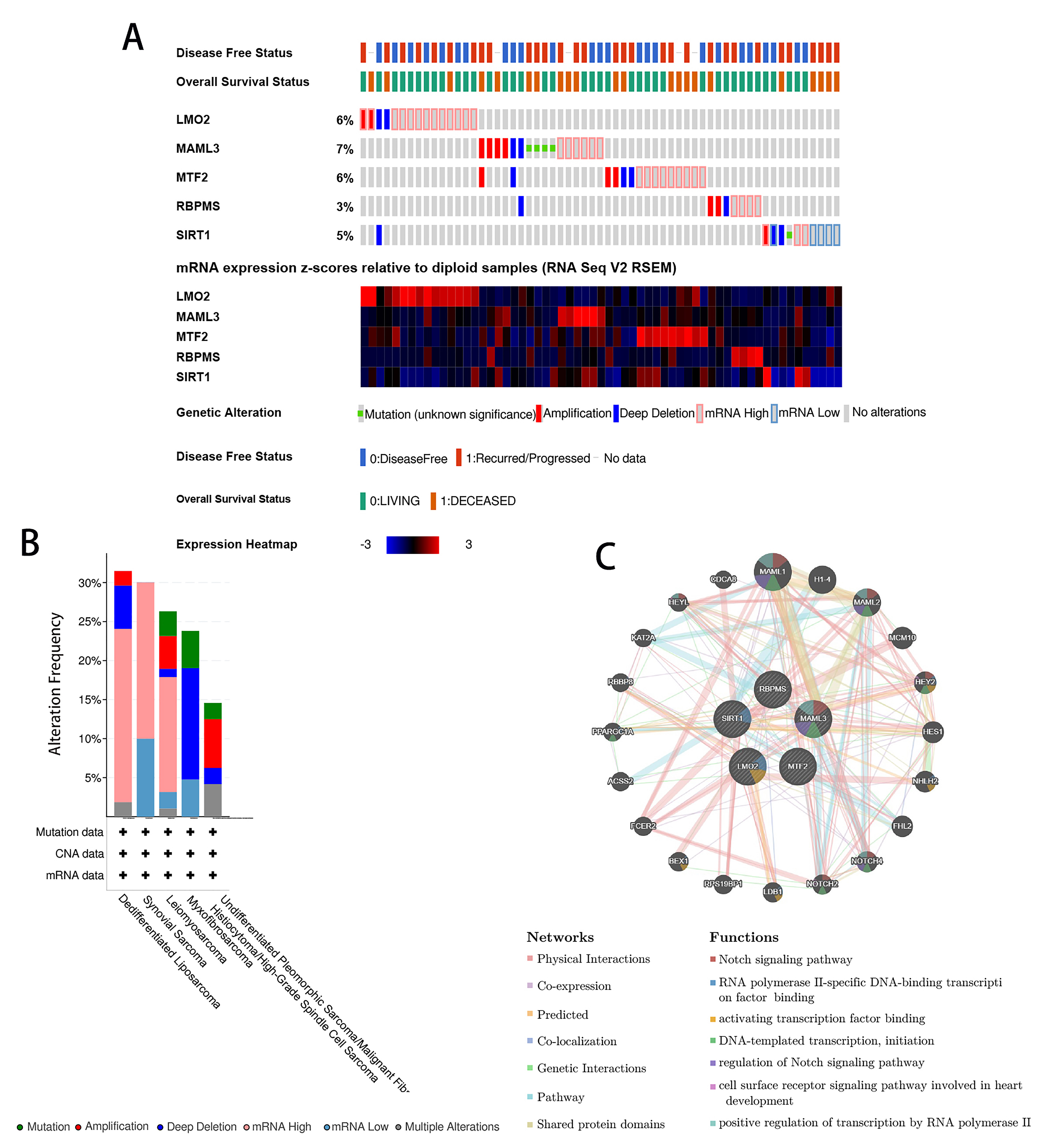


**Supplementary Figure S2.** Functional enrichment analysis and alteration of five genes in model. (A, B) Genetic variations of the 5 TcoF-related genes. (C) Functional enrichment analysis of the 5 TcoF-related genes.

**Supplementary Figure S3**





**Supplementary Figure S3.** Gene analysis of prognosis in patients with sarcoma. (A-E) The influence of 5 genes (LMO2, MAML3, MTF2, RBPMS and SIRT1) on overall survival in patients with sarcoma.

**Supplementary Figure S4**





**Supplementary Figure S4.** Gene analysis of prognosis in patients with sarcoma. (A-E) The influence of 5 genes (LMO2, MAML3, MTF2, RBPMS and SIRT1) on disease-free survival in patients with sarcoma.

**Supplementary Figure S5**


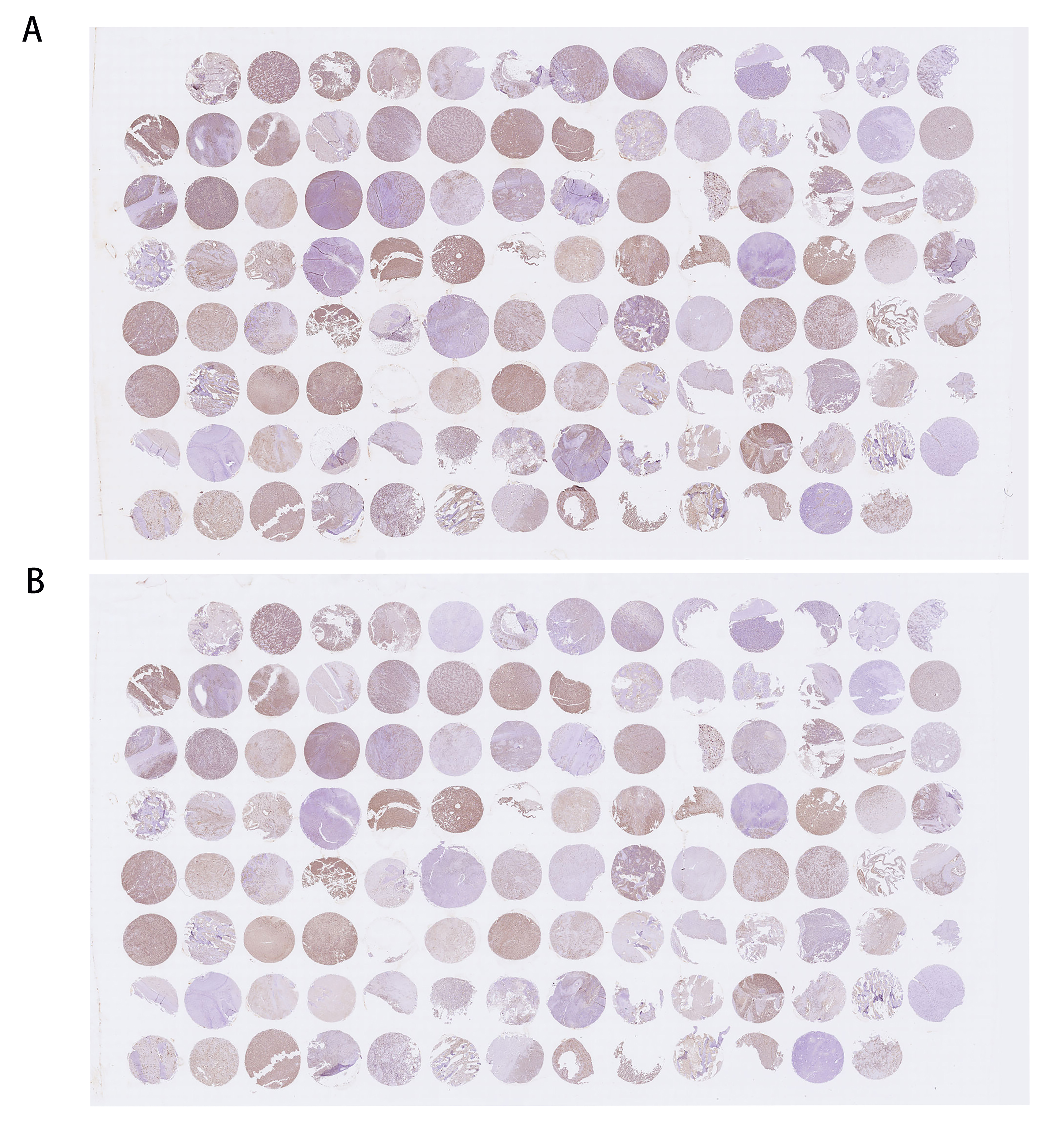


**Supplementary Figure S5** The results of TMA for 110 osteosarcoma tissues. (A) The image of MTF2 expression on TMA. (B) The image of RBPMS expression on TMA. TMA, tissue microarray.

**Supplementary Figure S6**





**Supplementary Figure S6** 13 TcoF-related genes prognostic risk score model. (A) Survival analysis to verify the prognostic model based on TARGET with 13 TcoF-related genes. (B) Survival analysis to verify the prognostic model based on GSE21257 with 13 TcoF-related genes.
